# Intracellular and tissue specific expression of FTO protein in pig: changes with age, energy intake and metabolic status

**DOI:** 10.1038/s41598-020-69856-5

**Published:** 2020-08-03

**Authors:** Karolina Ferenc, Tomaš Pilžys, Damian Garbicz, Michał Marcinkowski, Oleksandr Skorobogatov, Małgorzata Dylewska, Zdzisław Gajewski, Elżbieta Grzesiuk, Romuald Zabielski

**Affiliations:** 1 Veterinary Research Centre, Department of Large Animal Diseases and Clinic, Institute of Veterinary Medicine, Warsaw University of Life Sciences, Nowoursynowska 100, 02-797 Warsaw, Poland; 2 Institute of Biochemistry and Biophysics, Polish Academy of Sciences, Pawińskiego 5A, 02-106 Warsaw, Poland

**Keywords:** Proteins, Experimental organisms, Imaging, Microscopy, Animal physiology, Biochemistry, Biological techniques, Zoology, Diseases

## Abstract

Genome-wide association studies in the *FTO* gene have identified SNPs correlating with obesity and type 2 diabetes. In mice, lack of *Fto* function leads to intrauterine growth retardation and lean phenotype, whereas in human it is lethal. The aim of this study in a pig model was to determine the localization of the FTO protein in different tissues and cell compartments, in order to investigate potential targets of FTO action. To better understand physiological role of FTO protein, its expression was studied in pigs of different age, metabolic status and nutrition, using both microscopic methods and Western blot analysis. For the first time, FTO protein was found in vivo in the cytoplasm, of not all, but specific tissues and cells e.g. in the pancreatic β-cells. Abundant FTO protein expression was found in the cerebellum, salivary gland and kidney of adult pigs. No FTO protein expression was detected in blood, saliva, and bile, excluding its role in cell-to-cell communication. In the pancreas, FTO protein expression was positively associated with energy intake, whereas in the muscles it was strictly age-related. In IUGR piglets, FTO protein expression was much higher in the cerebellum and kidneys, as compared to normal birth body weight littermates. In conclusion, our data suggest that FTO protein may play a number of distinct, yet unknown intracellular functions due to its localization. Moreover, it may play a role in animal growth/development and metabolic state, although additional studies are necessary to clarify the detailed mechanism(s) of action.

## Introduction

Genome-wide association studies (GWAS) have shown that single nucleotide polymorphisms (SNPs) in intron 1 of the *FTO* (Fat mass and obesity associated) gene are strongly correlated with an increased risk of obesity in humans^1–4^. In the human *FTO* gene, the mutation (alteration p. Arg316 Gln) that inhibits catalytic activity of the protein, results in an autosomal recessive lethal syndrome^5^. In *Fto* knock-out mice, body weight and fat mass decrease^6^, while in animals overexpressing FTO they increase^7^. Evidence from genetic epidemiology studies, life-course modeling, and diet-induced fetal programming data suggests that the *FTO* gene plays an important role in these complex biological interactions. It may provide the missing link in the developmental regulation of energy metabolism. The *FTO* variants associated with intrauterine growth retardation (IUGR) and, in consequence, low birth weight, confer a predisposition to obesity later in life. This finding favors the hypothesis of the existence of a common genetic denominator that predisposes to low birth weight and obesity in adults^8–10^. Specifically, *Fto* deletion caused delayed growth, decreased white body fat, increased energy metabolism, and systemic sympathetic activation^6^. For example, in wild-type mice, fasting reduced *Fto* mRNA levels and the number of Fto-immunoreactive cells in the hypothalamus. Interestingly, glucose treatment reversed this effect^11^. However, another group of researchers showed that palatable sucrose feeding did not affect *Fto* expression in the mouse hypothalamus^12^. Further, Johannson and coworkers revealed that leucine intake increased *Fto* gene expression in hypothalamus^13^; however, the opposite effect was obtained by another group^12^. Other studies have shown that a high-fat diet increases *Fto* mRNA expression in white adipose cells^14^, as well as mRNA and protein levels in rodent liver^15^. Recently, it has been shown that increased carbohydrate and protein intake significantly up-regulates *FTO* mRNA in peripheral blood of adolescent boys; however, this also depends on *FTO* genotypes^16^. Further, *FTO* expression changes after intensive lifestyle intervention depended on *FTO* SNP rs9930506 type^17^. Finally, Yuzbashian and coworkers showed that changes in expression of *FTO* mRNA in visceral and subcutaneous adipose tissue depended on carbohydrate intake in humans^18^. Some results have suggested that SNPs in *FTO* gene might not influence obesity and diseases of affluence directly, but rather epigenetically impact the expression of neighboring genes: *IRX3* or *RPGIP1L*^19–21^. The strong genetic correlations between obesity and FTO were found about 12 years ago and the FTO protein has been crystallized^1,2,22^. However, thus far the occurrence of genetic variants and expression of *FTO* in correlation with obesity, diabetes, and cancer in different populations has been widely discussed^1,2,23–25^, but the understanding and characterization of the *product* of these genes—FTO protein is still poorly understood.

It is known that FTO protein belongs to a family of ALKBH non-heme Fe(II) and 2-oxoglutarate (2OG)-dependent oxidative DNA/RNA demethylases, homologs of bacterial AlkB protein^26^. FTO protein is typically localized in the nucleus^26,27^, but some researchers have observed both nuclear and cytoplasmic localization in cell lines^28,29^. The major physiological substrate of FTO is N6-methyladenosine (N^6^meA), abundantly present in RNA, while methylated thymine (3meT) in single stranded DNA (ssDNA) and uracil (3meU) in RNA are repaired much less efficiently^26,27^. The poor capability of FTO protein to repair DNA alkylation damage, as compared to other dioxygenases, suggests that this is not its main physiological role^22^. Studies in mice model indicate that there is a positive correlation between the expression of FTO protein and body weight^7^. It has been suggested that FTO may directly regulate food intake, fat development, energy metabolism, cell proliferation, and cancer development^30–38^. It has also been found that the availability of glucose and amino acids regulates FTO protein expression: glucose/amino acid starvation leads to a decrease in the level of FTO protein^39^. Thus, it is likely that the FTO protein is involved in the control of energy balance.

Summing up, the exact mechanism of the influence of nutrient intake on *FTO* gene expression is still unknown. Up until now, the published research regarding FTO has mainly focused on association between specific SNPs in *FTO* gene and high body mass index (BMI) or diseases of affluence. However, there are few papers showing the influence of nutrient intake on *FTO* gene expression. Further, thus far researchers have measured the level of *FTO* mRNA, rather than the level of the protein, and the experiments have been performed primarily in rodents and not species with metabolisms more similar to humans^40^ (Supplementary Figure S1).

Given the considerable sparsity of data concerning FTO protein and the lack of information regarding its expression in vivo in large mammals, we hypothesize that it is essential to establish the localization of FTO in tissues, in order to determine the physiological role of FTO protein in mammals, including humans. The strongest evidence for the need of such research is that in humans the deletion of *FTO* gene is lethal, which is not the case in rodents^5,6^. Pigs seem to be an optimal model to develop for these studies, due to the many similarities in the physiology and pathophysiology, including metabolic syndromes^41^, between pigs and humans. Thus, the present work is intended as an important first step towards understanding the role of FTO protein in large mammals. In order to address the present gap in the knowledge, the aim of this study was to determine FTO protein localization both in cell compartments, as well as in specific cells within pig tissues, in order to investigate potential FTO targets of action in the organism. Additionally, the impact of age, metabolic status, and energy intake on FTO levels was investigated.

## Results

### FTO SNPs sequencing

To determine homogeneity of the pig cohort with respect to the *FTO* gene, we investigated SNPs in Polish Landrace crossbreed with Duroc × Pietrain pigs (n = 29), similarly as performed in Danish commercial pigs and Gottingen minipigs by Madsen and coworkers^42^. We verified four *FTO* SNPs in exon 3 (213 A > G; 528 C > T; 594 C > G) and exon 6 (1,080 G > A). Table 1 summarizes the results concerning the frequency of the SNPs in these pigs. We did not find any differences in genotype association depending on groups, body weight, or blood parameters (data not shown).

**Table 1:** Frequency of alleles in SNPs in Polish Landrace 50% ♀ and Duroc 25%, Pietrain 25% ♂ pigs (n = 29).

Allel	Exon 3						Exon 6	
	213 A > G		528 C > T		594 C > G		1,080 G > A	
	n	%	n	%	n	%	n	%
A	4	14.3	–	–	–	–	–	–
G	6	21.4	–	–	6	21.4	29	100
C	–	–	28	100	4	14.3	–	–
Heterozygote	18	64.3	–	–	18	64.3	–	–

Results prepared accordingly Geneious 10.2.6 software.

### FTO localization in 11-month old pigs

**Figure 1:**
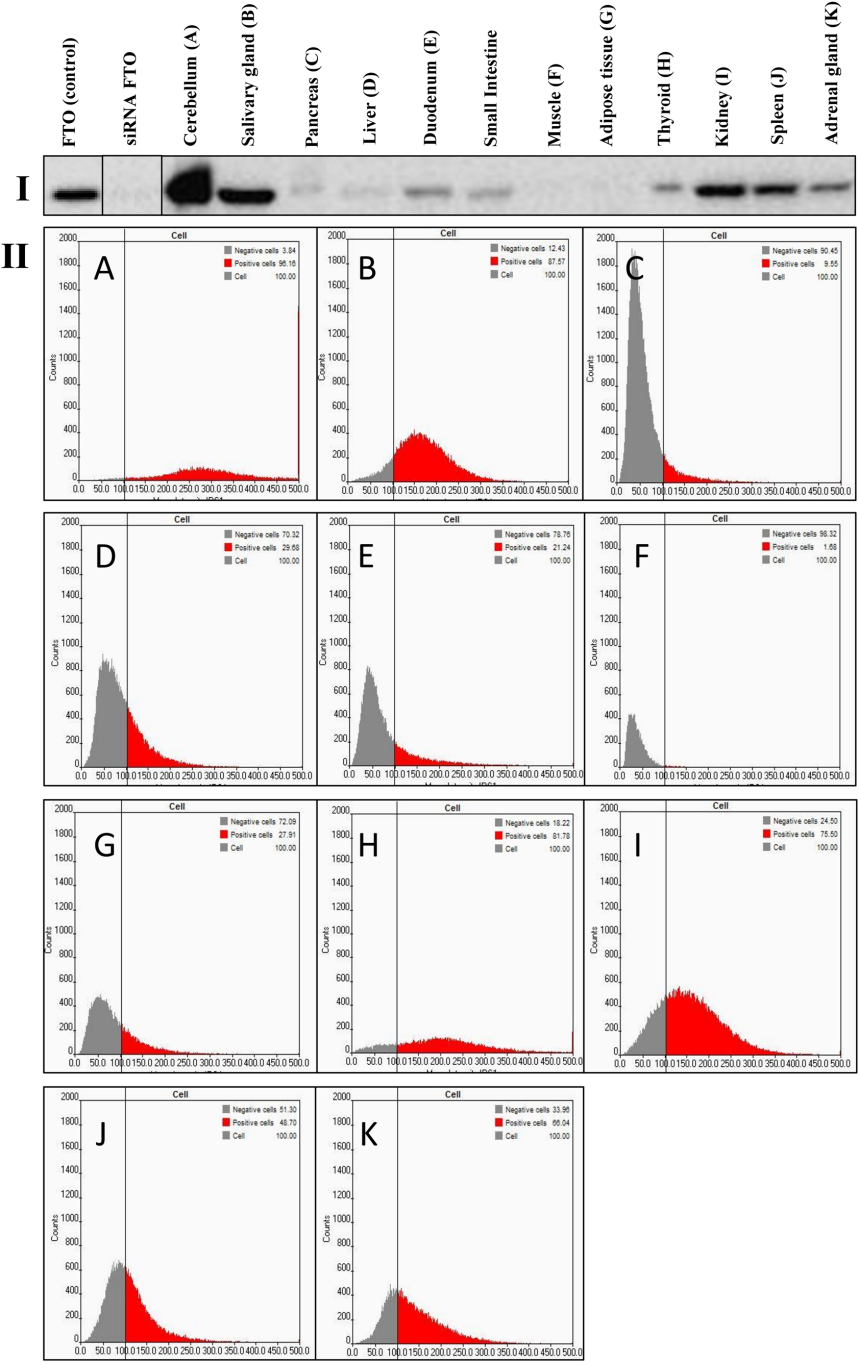
FTO protein expression levels in tissues of 11-month old pigs (n = 6). I—Western blot analysis, FTO (control)—HeLa cells not treated by siRNA, siRNA FTO—HeLa cells treated with siRNAs directed towards *FTO* gene (Image Lab, ChemiDoc MP Imaging System). II—in-tissue cytometry histograms of selected tissues (**A**–**K**) drawn by mean intensity of fluorescence (arbitrary units); percentage of high FTO expression (red—positive cells) is up to 200 arbitrary units (Scan^R and ScanR software, Olympus).

**Figure 2:**
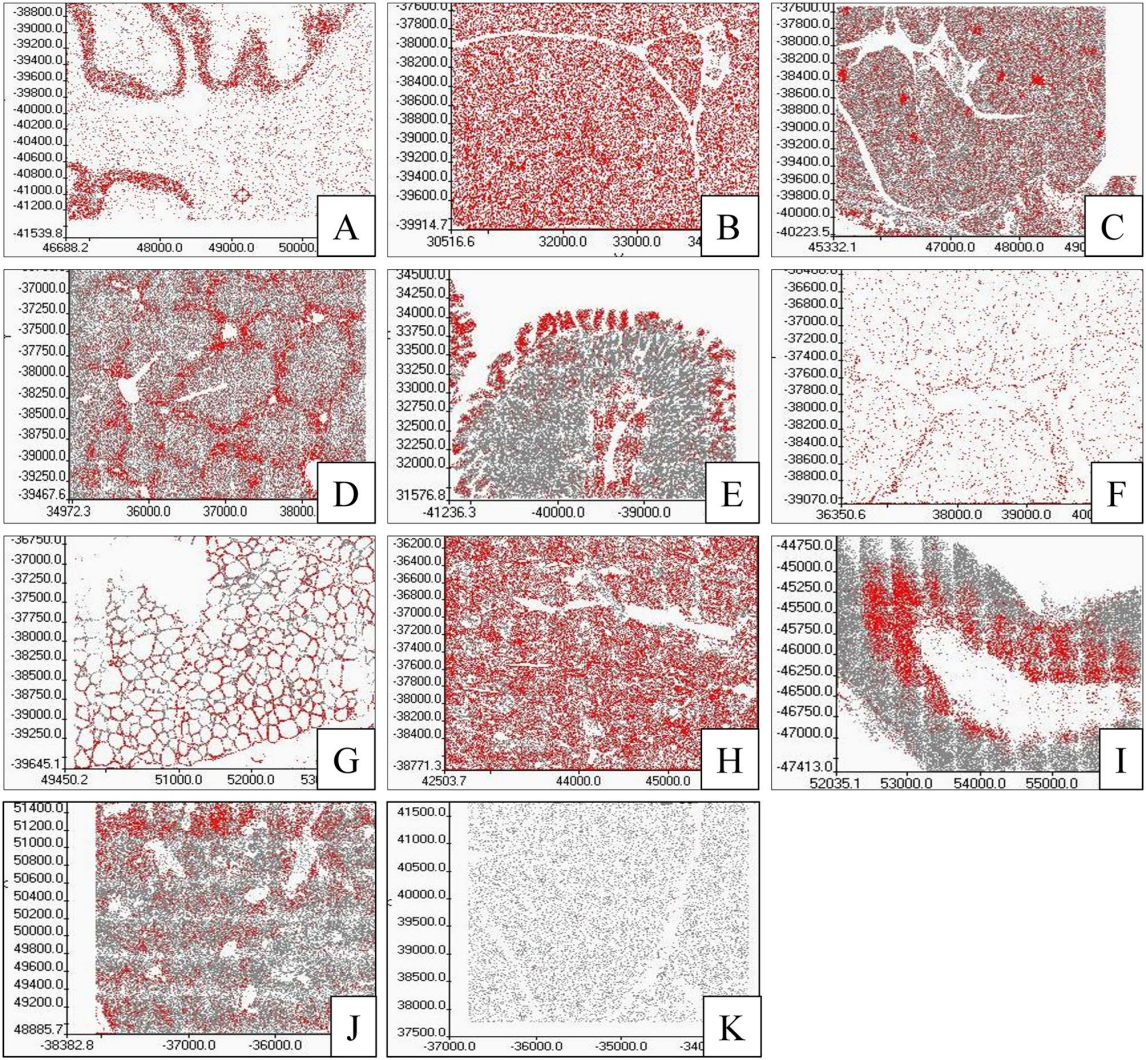
The map of specific regions of the FTO protein expression (red color) in pig tissues. (**A**) cerebellum, (**B**) salivary gland, (**C**) pancreas, (**D**) liver, (**E**) small intestine, (**F**) adipose tissue, (**G**) thyroid, (**H**) kidney, (**I**) adrenal gland, (**J**) spleen, (**K**) muscle. (Scan^R and Scan^R software, Olympus).

**Figure 3:**
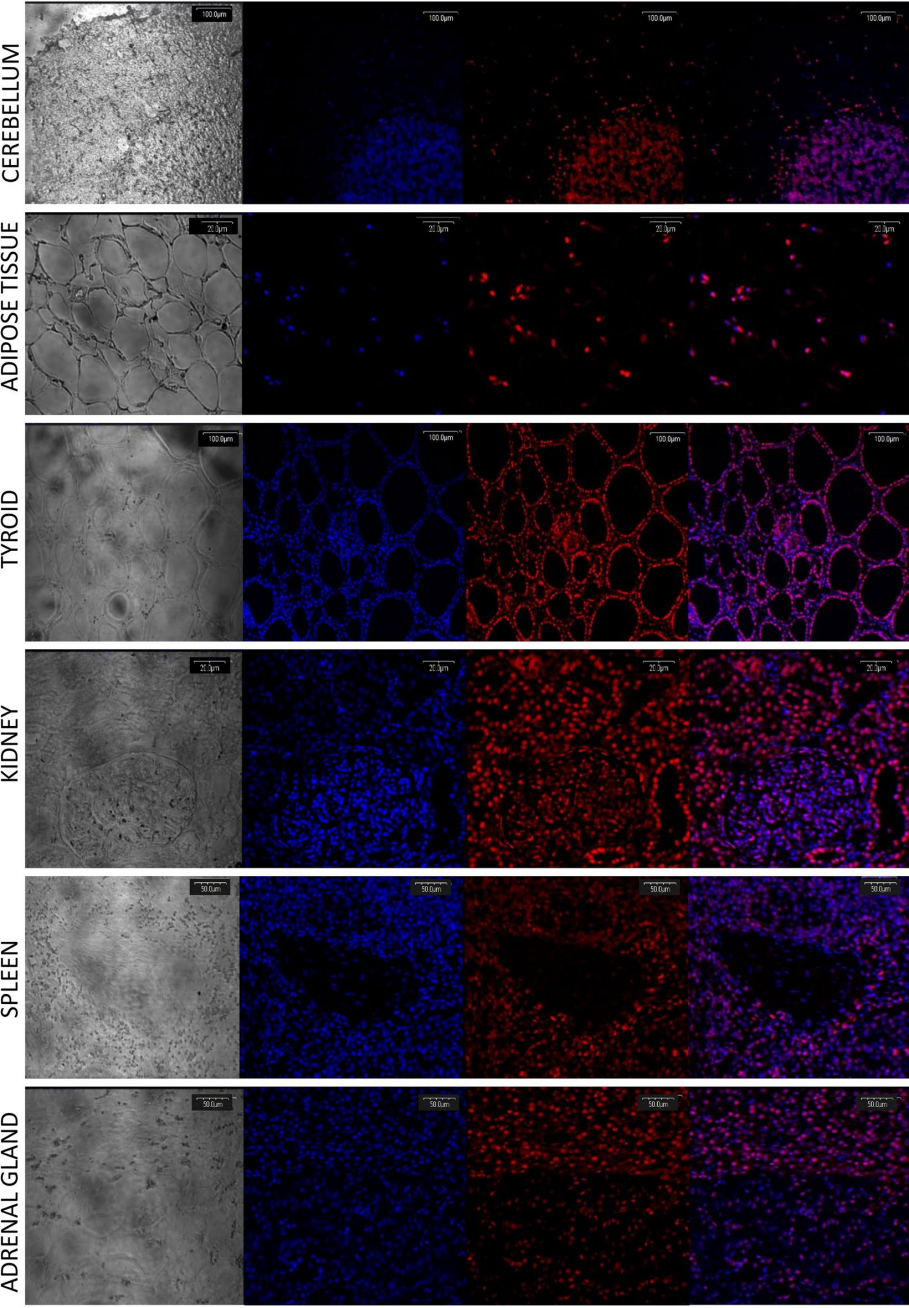
FTO protein expression in: cerebellum, adipose tissue, thyroid, kidney, spleen, and adrenal gland. Columns from the left: first—unstained view, captured using transparent channel; second—nuclei staining with Hoechst 3558, visualized as blue fluorescence; third—FTO expression, visualized by AlexaFluor 568 as red fluorescence; fourth—merged images of channels (Olympus FV500; Flouview 5 software, Olympus).

**Figure 4:**
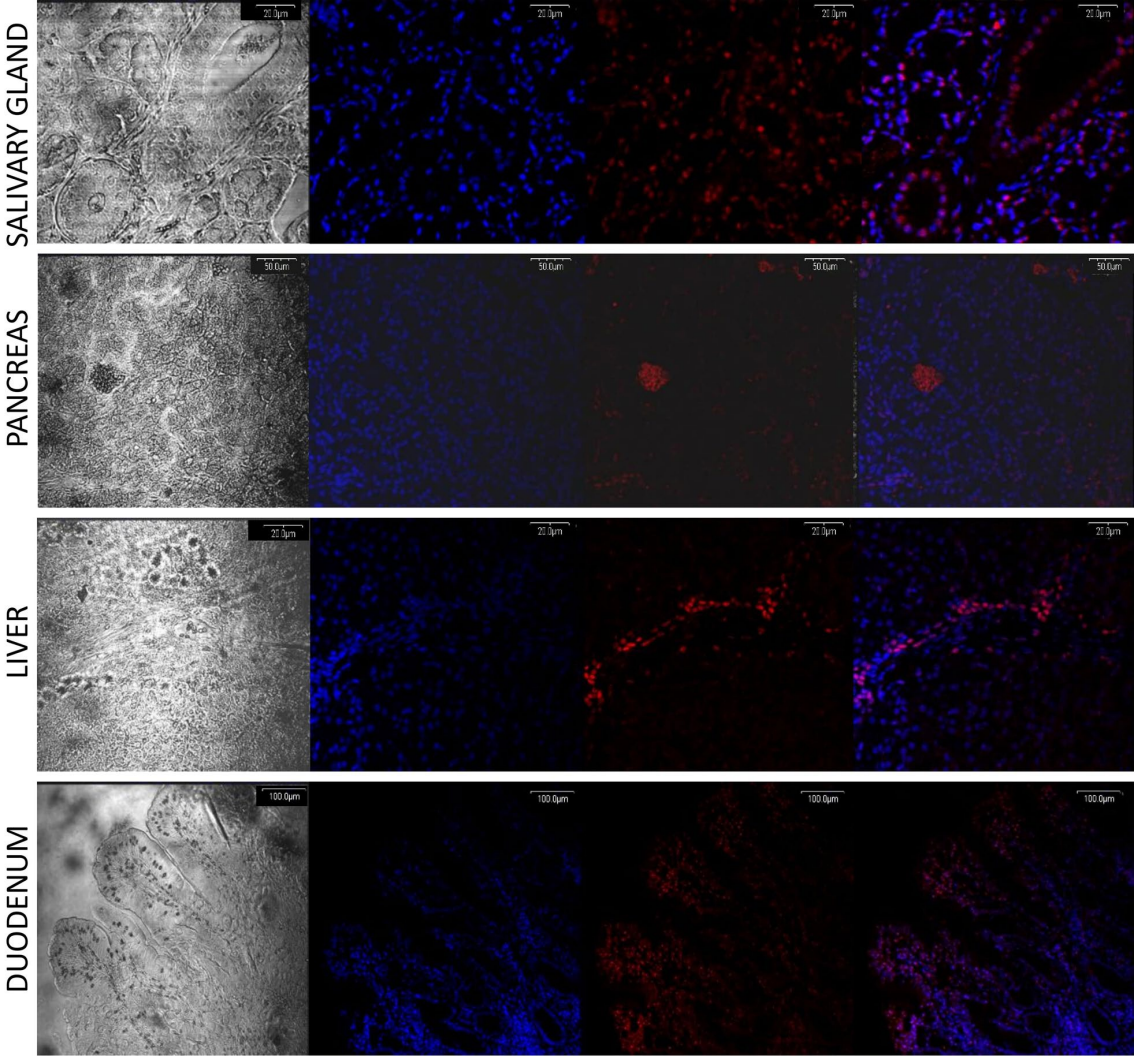
FTO protein expression in the tissues of gastrointestinal tracts. Columns from left: first—unstained view, captured using transparent channel; second—nuclei staining with Hoechst 3558, visualized as blue fluorescence; third—FTO expression, visualized by AlexaFluor 568 as red fluorescence; fourth—merged images of channels (Olympus FV500; Flouview 5 software, Olympus).

**Figure 5:**
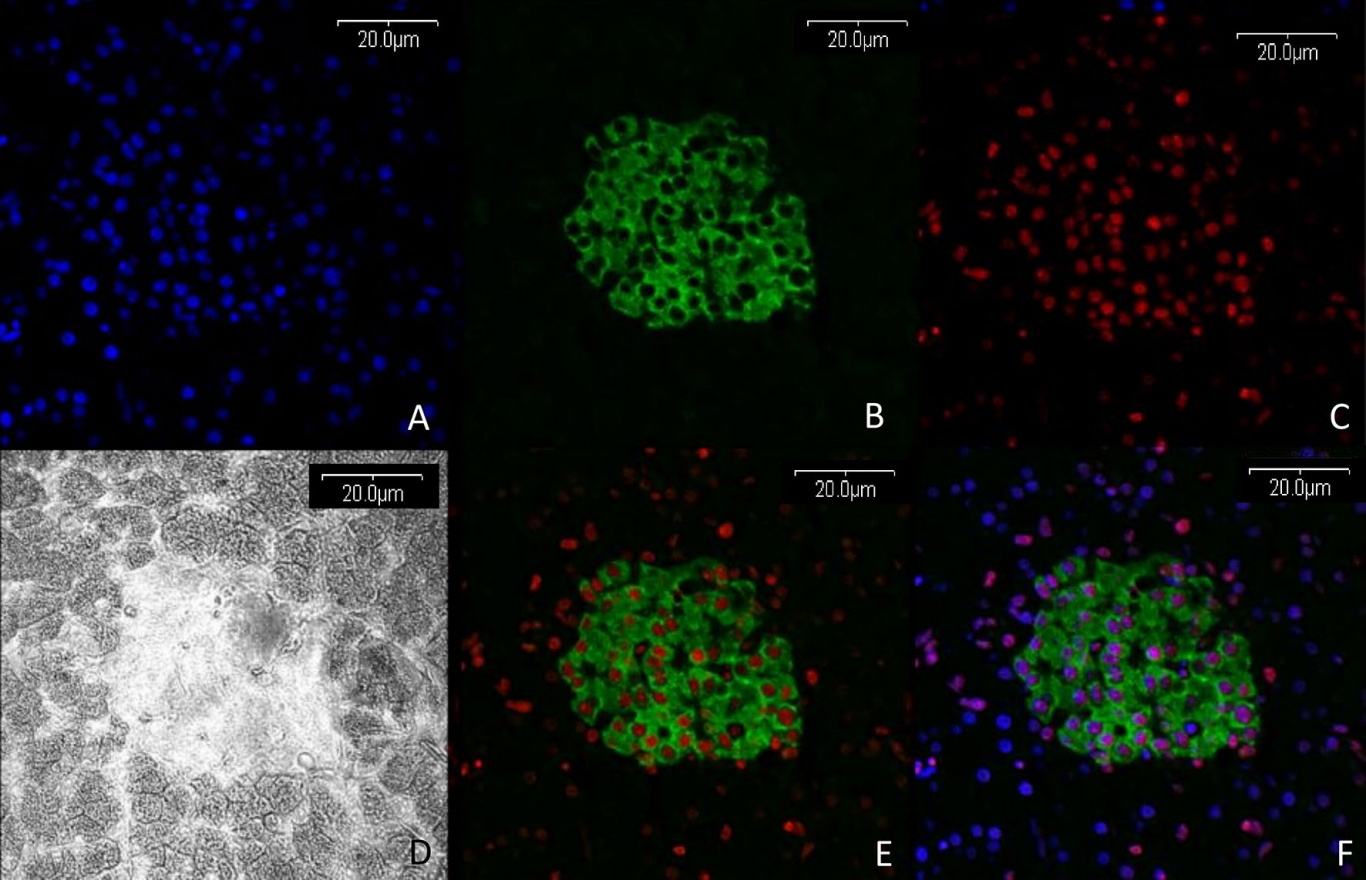
Colocalization of high expression of the FTO protein with insulin released in the beta cells within the Langerhans islets. Blue fluorescence—nuclei visualized by Hoechst 3558, green fluorescence—insulin visualized by secondary antibody AlexaFluor 488; red fluorescence—expression of the FTO protein visualized by secondary antibody conjugated with AlexaFluor 568. (**A**–**C**) single channels, (**D**) transparent view of unstained section, (**E**) colocalization of FTO with insulin, (**F**) merged images from channels A + B + C (Olympus FV500; Flouview 5 software, Olympus).

**Figure 6:**
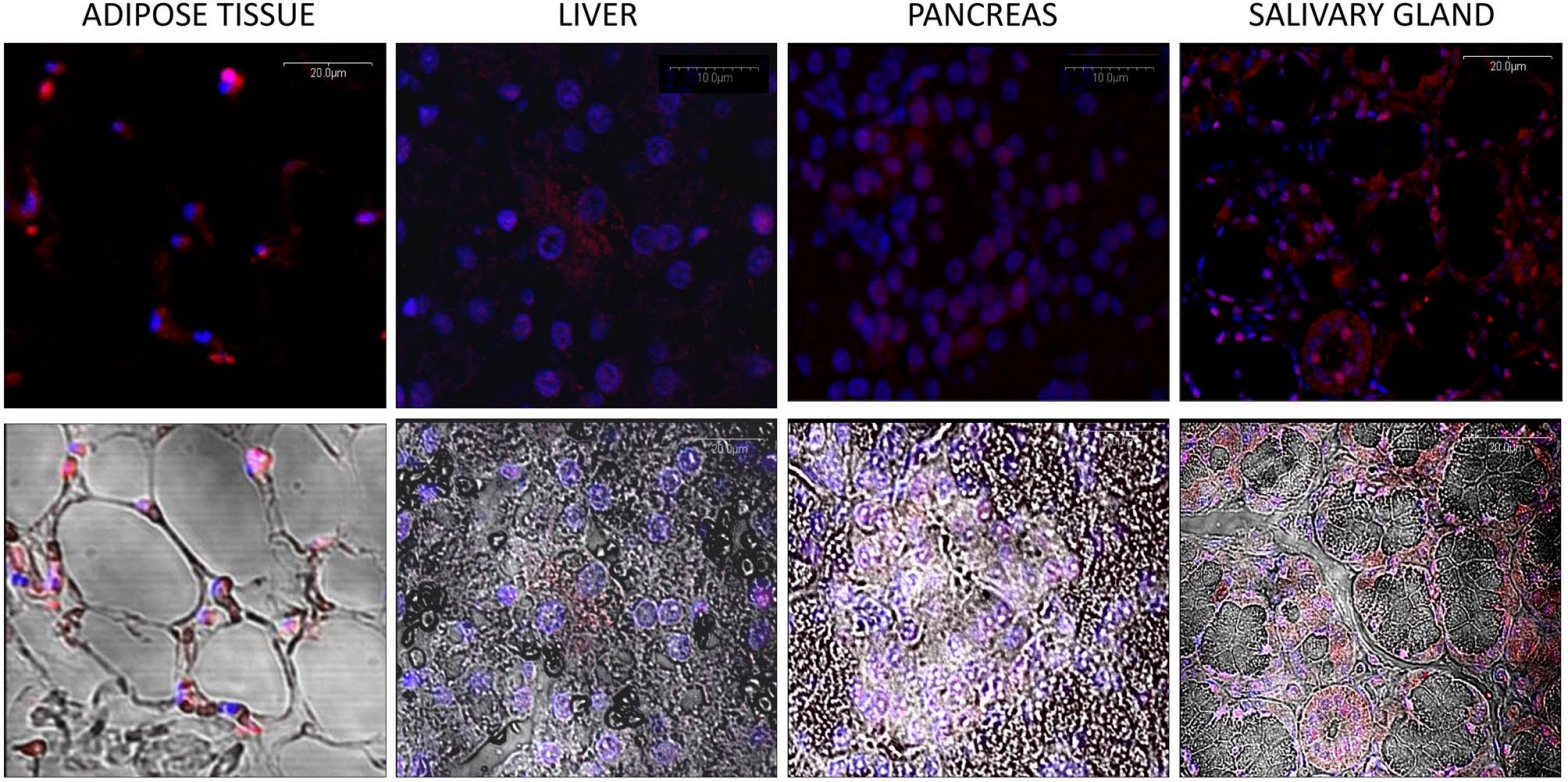
Cytoplasmic expression of the FTO protein in the adipose tissue (fat), liver, pancreas, and salivary gland. Top row—merged images from UV laser and green laser channel visualized nuclei (blue fluorescence) and FTO (red fluorescence). Bottom row—merged images from UV laser; green laser channel and transparent unstained view of the visualized nuclei (blue fluorescence); FTO (red fluorescence) and the structure of tissue (Olympus FV500; Flouview 5 software, Olympus).

To verify anti-FTO antibodies, the method of RNA interference directed toward FTO protein in the HeLa cell line was used, confirming proper antibody action (Fig. 1I, line FTO (control) vs. siRNA FTO, Supplementary Figure S1). In pigs fed with the control diet, Western blot analysis showed: high FTO expression in the cerebellum, salivary gland, kidney, and spleen; low expression in the duodenum, jejunum, thyroid, and adrenal gland; and the lowest expression in the pancreas, liver, skeletal muscles, and adipose tissue homogenates (Fig. 1I, Supplementary Figure S1). The average level of FTO protein in tissues was confirmed by the in-tissue cytometry method, where the structure of the tissue was maintained (Fig. 1II). Moreover, FTO expression maps created with the use of in-tissue-cytometry indicated specific areas in most of the tissues where the FTO expression was most abundant (Fig. 2). The histological tissue analysis (transparent view) and the analysis of the FTO expression as a marker using confocal microscopy identified particular types of cells where the expression of the FTO protein was dominant. In the cerebellum, high FTO expression was found in the spaces where cell density was highest, while no FTO content was observed in Purkinje cells (Fig. 3). In the salivary gland, the protein distribution was seemingly homogeneous (Fig. 4), but no FTO protein was found in the saliva (unpublished data). In the pancreas, a very high level of the FTO was found only in a few, chiefly insulin-producing pancreatic cells (Fig. 5). The localization of these cells coincides with the localization of the Langerhans islets (Figs. 2C and 4). In contrast, the FTO expression was very low in the pancreatic acinar and ductal cells (Fig. 4). In the liver, abundant FTO expression was observed in the hepatocytes located on the outskirts of the liver lobules and most abundantly in the cells surrounding the portal triads (small arteries, veins, and bile ducts). However, no FTO protein was detected in the bile. The hepatic lobule area, near the central vein, was also free of FTO protein, producing a clear-cut “halo effect” (Figs. 2D and 4). In the small intestine (duodenum and jejunum), high FTO levels were observed in the mucosa, especially in the apical part of the villi, as well as in the crypt region (Figs. 2E and 4). In adipose tissue and the thyroid gland, FTO protein was evenly distributed in the cells (Figs. 2F,G and 3). Measurements in adipose tissue were affected by the abundance of the fat vacuoles, because the FTO expression was quite high in the adipocyte cytoplasm and as many as 75% of adipocytes were FTO-positive. However, the FTO-free fat droplets, occupying major adipocyte space, markedly reduced the overall results of the counting (see Figs. 2F, 3, and 6). In the kidney, the level of FTO protein was generally high, apart from the Bowman’s capsules (Figs. 2H and 3). Meanwhile, in the spleen FTO protein was found only in the red pulp (Figs. 2J and 3). In the adrenal gland, the level of the protein differed between the cortex and the medulla (Figs. 2I and 3). Finally, no specific FTO expression was detected in skeletal muscles (Fig. 2K). Interestingly, we found that the nuclear versus extranuclear FTO localization was characteristic for specific tissues: cytoplasmic localization was observed in the cells of adipose tissue, the liver, pancreas, and salivary gland (Fig. 6), while in the remaining tissues FTO protein was localized only in the cell nuclei. Moreover, no FTO protein was observed in cell excretions, such as saliva and bile, nor in the circulating blood plasma.

### Age-related differences in the FTO protein expression

**Figure 7:**
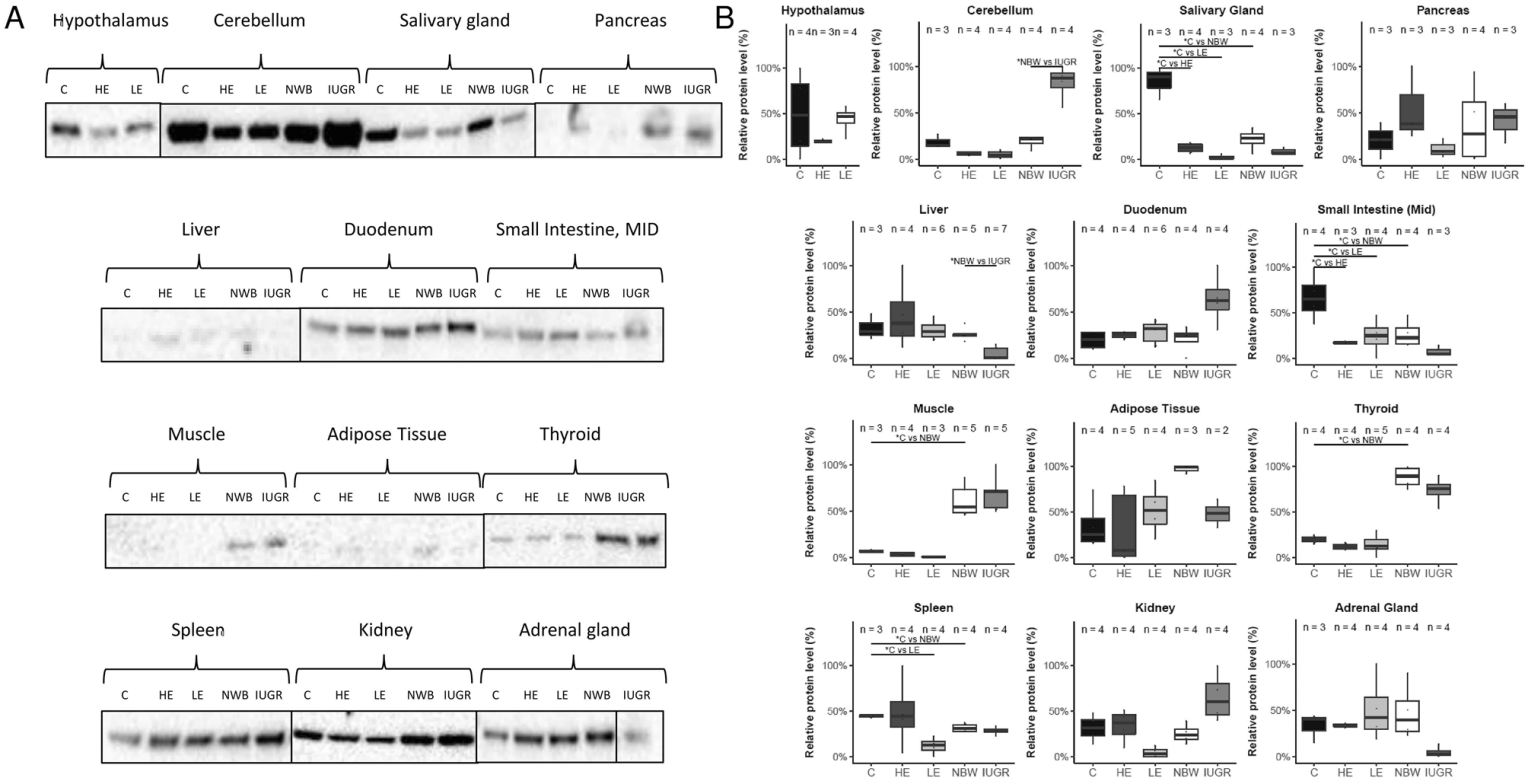
Western blot analysis of the FTO protein expression in pig tissues. (**A**) FTO band with protein load of 50 ng: (**C**) standard diet, HE—high energy diet with developed obesity and insulin resistance; LE—low energy diet as a model of chronic undernutrition; NWB—7-day old pig neonates with normal birth body weight; IUGR—intrauterine growth retarded pig neonates as a model for predisposition to obesity and T2D in adult life (Image Lab, ChemiDoc MP Imaging System). (**B**) Post-western blot densitometry analysis of the FTO level in pig tissues. (**C**) Standard diet (n = 6); HE—high energy diet with developed obesity and insulin resistance (n = 6); LE—low energy diet as a model of chronic undernutrition (n = 6); NBW—7-day old normal birth body weight pig neonates (n = 7); IUGR—7-day old pigs with intrauterine growth retardation (n = 7). *p < 0.05, two-tailed unpaired Student’s *t* test with Benjamini–Hochberg adjustment (software R version 3.3.0).

**Figure 8:**
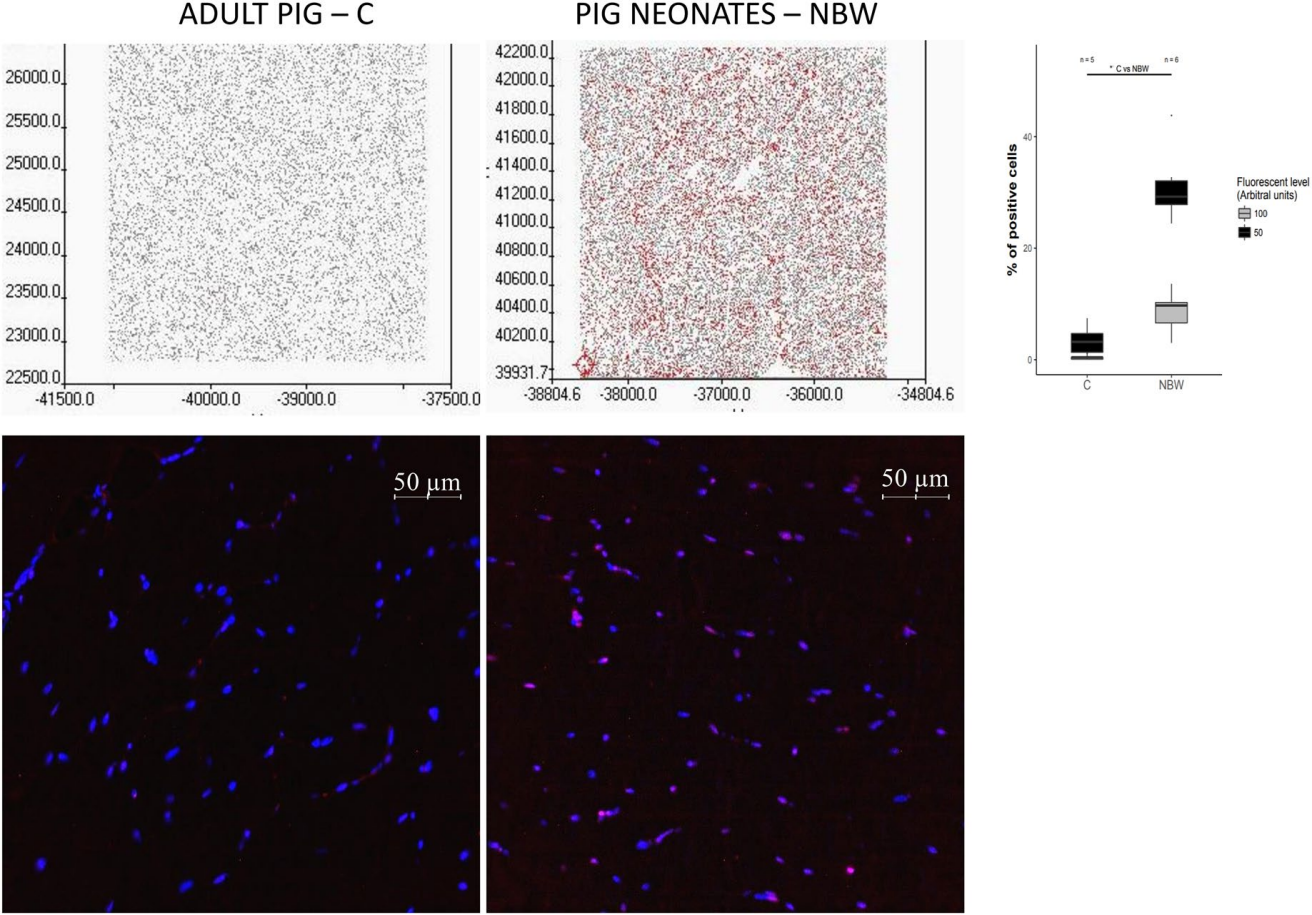
Differences in the FTO protein expression in skeletal muscles of adult and neonatal pigs. Top—representative FTO protein immunofluorescence visualization (left), and densitometry analysis (right). Bottom—representative maps with specific regions of high expression of the FTO protein (red color). *p < 0.05, two-tailed unpaired Student’s t-test with Benjamini–Hochberg adjustment. (**C**) 11-month old pigs, NBW—7-day old pig neonates (Scan^R and ScanR software, Olympus, software R version 3.3.0).

In comparison to 7-day old neonates with normal birth body weight (NBW), 11 months old pigs fed with the control diet (C) showed significantly higher expression of the FTO protein in the salivary gland (*p* value < 0.02), small intestine (*p* value < 0.05), and spleen (*p* value < 0.02). On the other hand, expression was lower in the thyroid gland (*p* value < 0.01) and there was a tendency toward lower expression in adipose tissue (*p* value = 0.054) (Fig. 7, Supplementary material S1). However, the most spectacular decrease in the level of FTO with age was found in the skeletal muscles (*p* value < 0.01 by Western blot and fluorescence) (Figs. 7 and 8, Supplementary material S1). Finally, FTO protein expression did not show any significant age-related changes in the cerebellum, pancreas, liver, duodenum, kidney, and adrenal gland.

### FTO protein expression in intrauterine growth retarded pig neonates (IUGR)

In comparison to NBW, the IUGR phenotype resulted in different expression of FTO protein in several tissues, (Fig. 7, Supplementary material S1). In the IUGR animals, FTO expression was higher in the cerebellum (*p* value < 0.02) and slightly higher in the kidney (*p* value < 0.08), as compared to NBW piglets. Conversely, the FTO protein expression was lower in the liver (*p* value < 0.01) and slightly lower in the salivary gland (*p* value < 0.09) and small intestine (*p* value < 0.07) in the IUGR neonates than in the NBW animals.

### FTO protein expression in pigs chronically exposed to low / high energy diet

**Figure 9:**
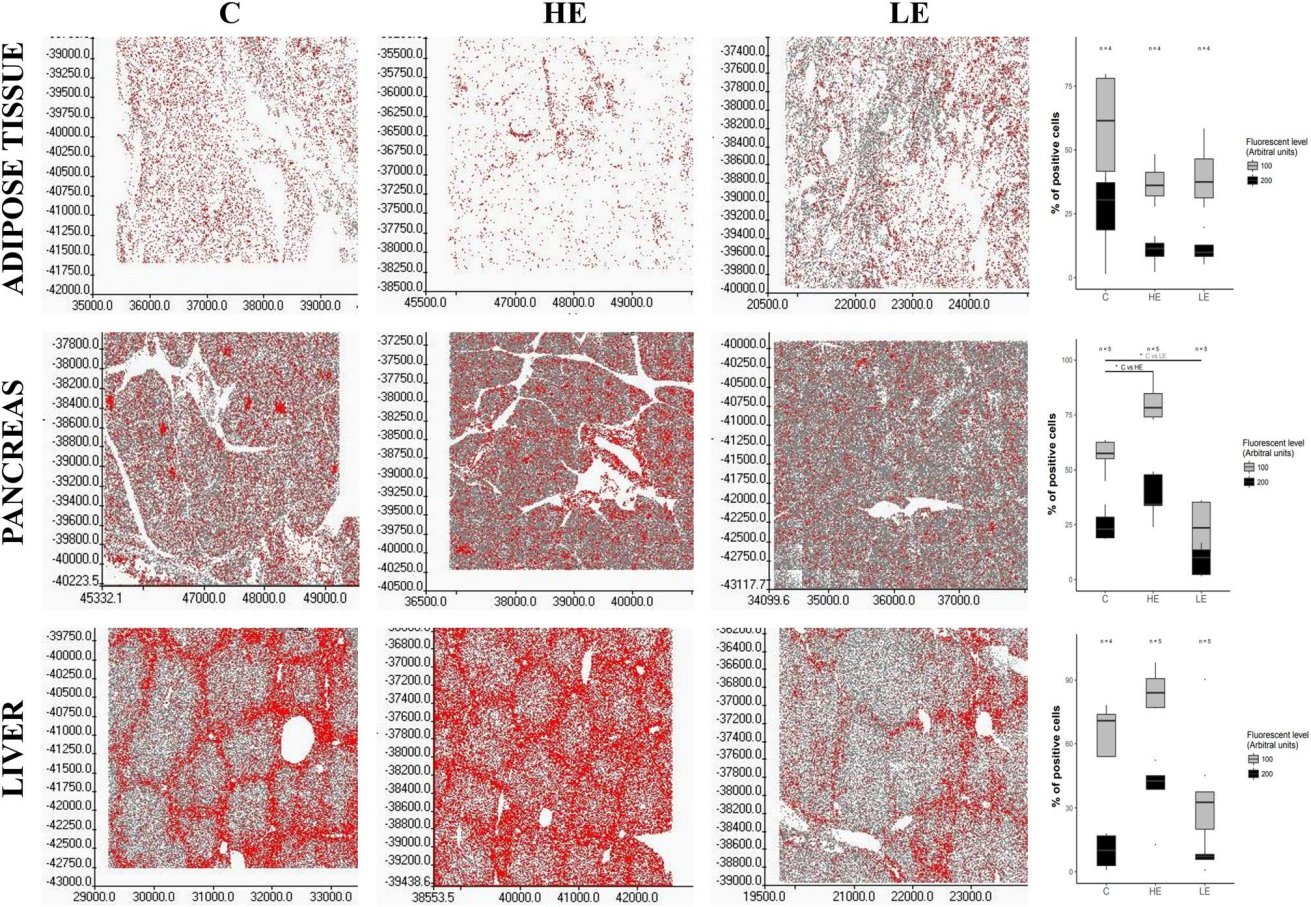
Representative maps of specific regions of the FTO protein expression (red color) in adipose tissue, pancreas, and liver of pigs fed a standard (C), high energy (HE) diet or low energy (LE) diet (left part). Right part shows densitometry analysis of FTO in the same tissues (n = 6). *p < 0.05, two-tailed unpaired Student’s t-test with Benjamini–Hochberg adjustment (Scan^R and ScanR software, Olympus, software R version 3.3.0).

**Figure 10:**
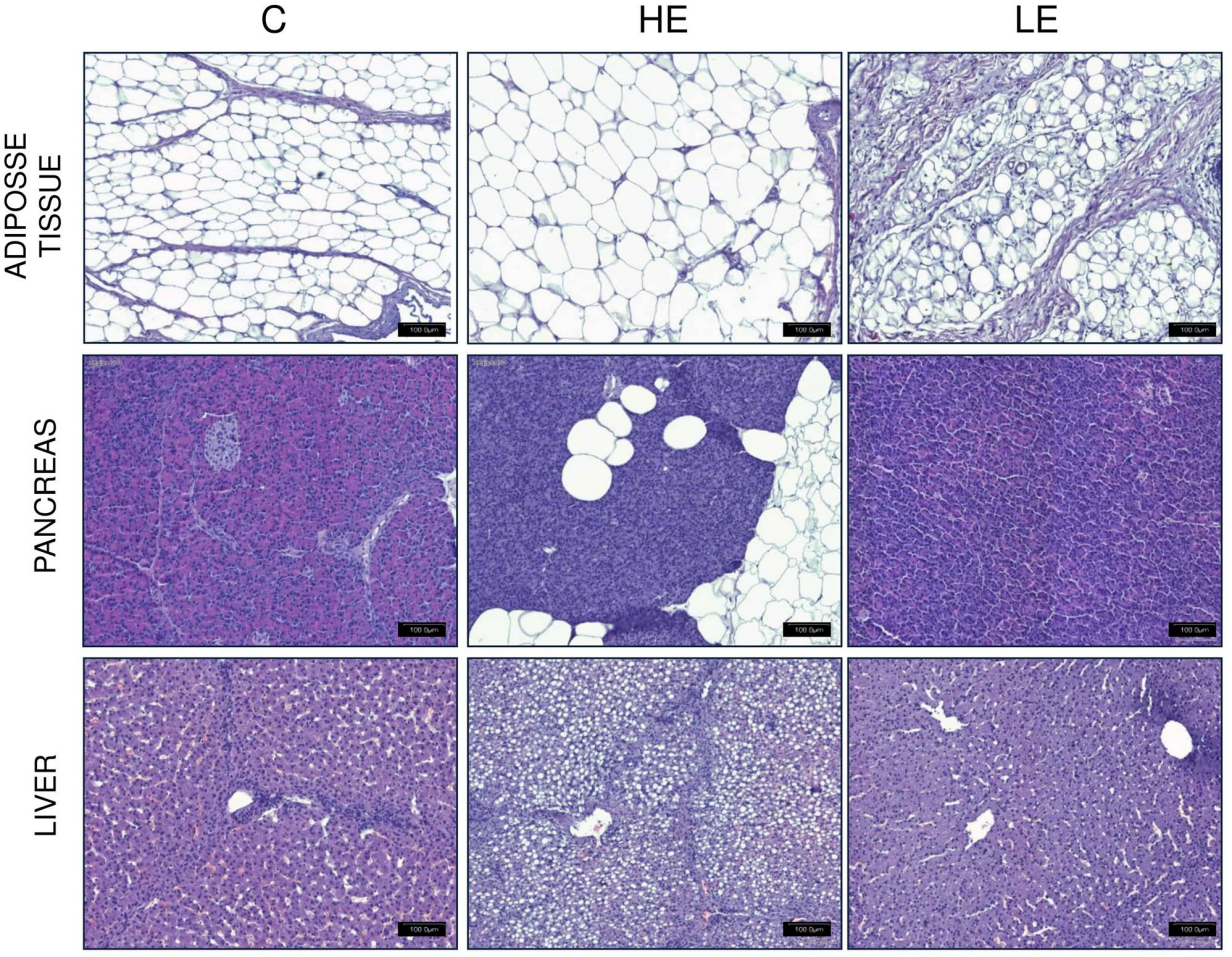
Histological images from adipose tissue, pancreas and liver of piglets fed a control, HE, or LE diet. The HE diet resulted in accumulation of fat droplets in the adipose tissue, as well as in the pancreas and the liver. The size of the Langerhans islets in the pancreases of pigs fed the LE diet. Hematoxylin and eosin staining (Olympus BX43 with cellSens Stanard 1.9. software; Olympus).

An imbalance in terms of energy (low energy diet—LE, 50% of the control nutritional energy and high energy diet—HE, 150% of the control nutritional energy) in the diet severely affected the structure of tissues and the adipocyte content (Figs. 9, 10). The changes were most profound in the liver and the pancreas: with the HE diet, adipocytes were abundantly present in the histological view of these tissues (Fig. 10).

In the majority of the examined porcine tissues, changes in FTO expression in response to either HE diet (leading to insulin resistance) or LE diet (leading to significant growth retardation) were uniform (Fig. 7, Supplementary material S1). Namely, according to Western blot analysis (Fig. 7, Supplementary material S1), there was a notably uniform reduction in FTO expression in three tissues: the cerebellum (*p* value < 0.1), salivary glands (*p* value < 0.02), and the small intestine (*p* value < 0.05) as compared to the controls. The LE diet led to significant reduced, or a tendency towards reduced, FTO expression in the spleen and kidneys (Fig. 7, Supplementary material S1; *p* value < 0.02 and < 0.1 respectively). We also observed a tendency towards lower FTO protein expression in the thyroid (*p* < 0.08) in the pigs fed the HE diet, as compared to controls. Additionally, it is worth mentioning that pigs fed the HE diet showed high level of variance in total mass-approx. fivefold higher than in the rest of the groups. This may be the reason for the large differences in FTO levels in adipose tissue, which is directly affected by the diet.

Homogenates of the tissues crucial for the development of obesity and Type 2 diabetes (T2D) contained very low levels of the FTO protein. As a result, we also analyzed FTO protein expression using in-tissue-cytometry. This method demonstrated a significant increase in pancreatic FTO expression in pigs fed the HE diet (*p* value < 0.01) and a reduction in pancreatic FTO expression in pigs fed the LE diet (*p* value < 0.01, Fig. 9). The latter finding corresponds well to the reduced number and size of the Langerhans islets in the pancreases collected from pigs fed the LE diet (Fig. 10). In addition to the Langerhans islets, abundant FTO expression was observed also in pancreatic acini of pigs fed the HE diet. In single images, we also observed higher FTO expression in the livers of the animals fed the HE diet (i.e. thicker “halo”) than in the livers of the control animals, albeit without statistical significance (Fig. 9).

## Discussion

In humans, some *FTO* SNPs are strongly associated with a predisposition to obesity. This association was the strongest of all known genetic changes^43^. In recent years, a few studies have been published on porcine SNPs in the *FTO* gene. Interestingly, an association of SNPs with intramuscular fat deposition has been found in pigs such as the Italian Duroc, the Danish commercial pig, as well as the Gottingen minipig^4,42,44–46^. In our study, we examined four SNPs, published earlier by Madsen and coworkers^42^, in 29 Polish Landrace × Duroc, Pietrain crossbreed pigs. We found that in the Polish Landrace crossbreed with Duroc × Pietrain, for SNP 594 C > G, 86% of pigs had the SNP (CG or GG) predisposing to increased amount of lumbar back fat, total percentage of lipids, and intramuscular fatness^4^. This may explain why we were successful in obtaining a high level of fatness and observed the development of insulin resistance in the group of pigs fed the HE diet. No heterozygotes, characteristic for Gottingen minipigs, for the SNPs 528 C > T and 1,080 G > A were found in our pigs. In all of the examined SNPs, we found only synonymous changes. As suggested by Madsen and coworkers, the nonsynonymous alleles were probably eliminated from commercial pig breeds by the selection pressure of intensive production^42^. In our experiments, in accordance with the results of *FTO* genotyping, we found that changes in FTO protein levels did not depend on genetic *FTO* variants. Instead, as was shown previously, factors such as a diet and lifestyle may directly impact the level of *FTO* expression^17^. In this work we decided to study whether-and how-age, metabolic status (normal birth weight vs. IUGR), and energy intake (low vs. high diet) directly influence FTO protein expression.

The distribution of *Fto* mRNA and protein levels was previously studied by several research groups, primarily using rodent models^26,29,31,42,47–49^. The role of *Fto* in rodents is, however, limited as compared to humans. In humans, mutations in the *FTO* gene lead to death before the third year of life. In rodents, *Fto* deletion does cause visible phenotype changes, but is not lethal^5,6^. Thus, the results of our research, based on a pig model, are much more translatable to human physiology than results obtained in rodents. Our study should provide a better understanding of the role of the human FTO protein. Furthermore, our FTO distribution study in pigs, conducted with the use of in-tissue cytometry and confocal microscopy, revealed that, even in the several tissues where FTO protein is expressed at low levels, its expression is linked to specific cell types. For example, in the pancreas expression of FTO is low in the exocrine part, but high in the Langerhans islets, chiefly in the insulin-producing cells. Abundant FTO protein expression could also be masked in some cells; for example, the presence of large fat droplets in adipocytes masked high FTO levels in their cytoplasm. Meanwhile, in the small intestine, FTO protein was expressed almost exclusively in the mucosa, chiefly in crypts, as well as in the upper part of the villi. This localization suggests a role of for FTO protein in controlling epithelial cell proliferation/differentiation and nutrient absorption.

In prior rodent studies, the highest *Fto* mRNA levels were observed in the hypothalamus, but we found that in our pigs the cerebellum was the richest source of FTO protein. This result is consistent with the results published on adult Danish commercial pigs by Madsen and coworkers^42^. In the present study, we also found that the salivary gland and kidneys had high expression of FTO protein. The abundance of FTO protein in kidney tissue suggests that it may play role in water and/or electrolyte reabsorption, rather than in filtration, because FTO protein was notably absent in Bowman’s capsules. In the salivary gland FTO may also play a role in electrolyte/water regulation, like in the kidney, through the control of serous salivary fluid formation, rather than a role in salivary enzymes production. This is also consistent with the very low FTO protein levels in the pancreatic acini that synthesize and secrete pancreatic digestive enzymes. The cause of high level of FTO protein in salivary cells might be intensive production of the extracellular proteins in this cell type. FTO may take a part in mRNA modification through demethylation of N^6^-meA in specific transcripts, thereby the protein can influence the stability and processing of transcript and the level of gene expression^36,50,51^.

The results of our tissue localization experiments have confirmed—for the first time in vivo—that FTO is expressed in the cytoplasm. Previously, data from several groups working with isolated cell models in vitro suggested cytoplasmic FTO localization^28,29^. For example, Aas and coworkers demonstrated endogenous FTO presence in the cytosol, membranes, and nuclear fractions of human U2OS cells^29^. Meanwhile, earlier reports showed that the FTO protein shuttles between the nucleus and cytoplasm^28^. However, our in vivo findings provide particularly important new information; we found that cytoplasmic localization is observed only in the cells of particular tissues and organs that had high overall FTO cellular expression (Fig. 6), namely in adipose tissue, the salivary gland, liver (hepatocytes adjacent to hepatic triads) and pancreas (β-cells). These findings suggest that in the cytoplasm of these tissues, FTO takes a part in the modification of nucleoprotein complexes. Interestingly, no FTO protein was detected in blood plasma nor in cell excretions, such as bile and saliva, indicating that FTO protein probably acts just locally inside the cell and does not take a part in cell-to-cell communication, as for example hormones and cytokines do.

FTO protein is produced already at the embryonic stage^5^, but *FTO* mRNA has also been found in the placenta^52,53^. Nevertheless, its function during embryonic and fetal development remains unknown. Moreover, *FTO* mRNA expression was found to be positively correlated with fetal or neonatal weight, suggesting some as-yet-unidentified role in fetal growth. Previously, in the cerebellum, Madsen and coworkers found higher expression of *FTO* transcripts at early gestation (day 50) than in late pregnancy (day 100) and in adult pigs^42^. Here, we suggest that FTO protein may play an important role not only in prenatal development, when it probably is involved in neurogenesis and weight control, but also in postnatal development. If we compare 7-day old piglets to 11-month old pigs, we observe relatively high levels of FTO protein in organs that develop more intensively just after birth, such as muscles, adipose tissue, and the thyroid. According to in vitro studies, at this stage of growth FTO protein may be involved in adipogenesis. Previous studies have also indicated that elevated expression of FTO protein in porcine preadipocytes takes a part in the promotion of cell proliferation, differentiation, and lipid deposition^31,54^, as well as in myoblast differentiation^55^. Comparing neonates with adult pigs, we detected FTO protein only in young pigs. Also it has been shown that up-regulation of *FTO* gene expression was associated with increase in skeletal muscle mass in adolescents, suggesting that FTO levels in skeletal muscle may be directly linked with growth of this organ^56^.

These changes may be linked to the extremely intensive development of the above-mentioned organs that occurs only in the early stages of postnatal life, because piglets usually double their birth weight within the first week of life. This is consistent with our previous discovery that seven out of nine ALKBH proteins, including FTO, are overexpressed in fast proliferating cancer cells^38^.

Thus, we propose that high demethylation efficiency, which demands expression of FTO protein, is crucial for the fine-tuning of gene expression at particular stages of pig development, such as just after birth. However, in the spleen, salivary gland, and in the small intestine, we observed the opposite effect: 11-month old pigs showed higher expression of FTO protein than neonates—and these organs also undergo severe tissue rebuilding and growth in the early postnatal period. Interestingly, we did not detect FTO protein expression in skeletal muscles of adult pigs. By comparison, Madsen and coworkers noticed relatively low expression of the *FTO* gene in 6-month old commercial Danish pigs, but these animals were still at the stage of intensive growth and protein deposition^42^. In our 11-month old pigs, intensive muscle development had already completed and the energy sources started to accumulate primarily in adipose tissue. Interestingly, we actually found the highest FTO protein expression in tissues not developing intensively after birth, which may suggest that in these organs/tissues FTO protein plays a role in maintaining homeostasis throughout life.

Due to altered fetal programming, IUGR syndrome may lead to higher predisposition to obesity and T2D in adult life, as compared to normal birth weight individuals. Molecular symptoms can be found even at early stages of postnatal life, at least in the small intestine and liver^57–59^. Further, previous results have shown that, both in rodents and humans, mutations in the *FTO* gene are linked with severe growth retardation^60^. For example, the R316Q mutation in human *FTO* leads to numerous severe phenotypes (including IUGR) and developmental delays, with all patients dying before the age of three^5^. As a result, we decided to examine the influence of the IUGR syndrome on levels of FTO protein. It has been postulated that FTO might be an "amino-acid sensor"^39^ in the organism and that one of the most important causes of intrauterine perturbations is an imbalanced maternal diet, especially one linked to changes in protein content^61^. Also there is some evidence that changes in amino acid transport through the placenta can lead directly to the creation of IUGR syndrome^40^. However, we did not find an unequivocal explanation for the divergent results in different tissues. In some tissues, such as the cerebellum and kidneys, FTO protein expression was much higher in IUGR piglets than in their normal body weight littermates, but in the salivary gland, small intestine, and liver expression was significantly lower. We speculate that the increased/decreased levels of FTO may be the result of altered methylation/demethylation levels in IUGR, as has been shown in several studies^30,62,63^, and that in the gastrointestinal system this may reflect retarded tissue structure and function development^57,59^. We conclude, that more research in this area is required.

Nowadays, the hypothesis that FTO acts as a sensor of amino acid concentrations under the regulatory effect of the mammalian target of rapamycin (mTORC1) has been verified^64^. However, the consequences of FTO activation by amino acids and its direct link with the mTOR pathways in vivo have yet to be proved. Clearly, the elucidation of the role of FTO in cell growth and differentiation is fundamental for the deciphering of its function in early development. The question of whether the sensing of amino acids by the FTO plays a direct role in the regulation of body weight, fat mass, and/or energy balance in vivo remains to be answered. However, the reduced FTO protein expression that we observed in the intestinal mucosa and liver of our piglets with IUGR syndrome supports this hypothesis.

The expression of *Fto* mRNA in the hypothalamic arcuate nucleus is bidirectionally regulated as a function of nutritional status. In rodent models, it has been show to decrease following a 48-h fast and increase after a 10-week exposure to a high-fat diet^26,35^. Likewise, in a sheep model it has been shown that decreased daily energy intake results in changes in *FTO* gene expression in the hypothalamus^53^. In our study, neither high nor low energy diets changed the level of FTO protein in the hypothalamus, according to Western blot analysis. However, in this experiment, the standard deviations for measurements of the control group were high and the number of animals used was limited. Nevertheless, we found diet-dependent changes in FTO levels in some peripheral tissues. One of our main discovery was that FTO protein levels in the pancreas were associated with energy intake: low on low energy diet and high on high energy diet, compared to normal diet. Earlier in vitro studies showed that FTO overexpression in MIN6 (mouse insulinoma) cells significantly inhibited insulin secretion in the presence of glucose, but did not affect insulin expression^47^. As a result, we argue that increased FTO levels in the pancreas may directly influence insulin release from beta cells. Moreover, FTO overexpression in the pancreas resulting from a high energy diet might also be a consequence of the reactive oxygen species (ROS) production and activation of the NF-κB pathway^47^. This hypothesis is plausible, because at early stages of T2D, pancreatic secretion is increased in response to the decreased number of insulin receptors in peripheral tissues and increased basal level of glucose in the blood^47^. The lack of a negative feedback signal forces the pancreas to increase insulin release, resulting in elevated levels of ROS production in the pancreas^47^. This may explain why we observed an increased FTO protein levels not only in β-cells, but also in the exocrine pancreas in pigs fed the HE diet. In this case, FTO could protect the pancreas against ROS and an insulin overload, because high insulin levels may lead to organ malfunction. Similar findings were obtained in the liver, where FTO was expressed more abundantly in cells close to the portal triads, and expression seemed to be associated with dietary caloric load.

Another interesting finding within this pool of experiments was that the LE diet influenced FTO expression more strongly than the HE diet. With the LE diet, decreased FTO expression was observed in several tissues, such as the salivary gland, small intestine, and spleen. Additionally, we observed that the kidneys tended to show lower levels of FTO. These changes may be linked to another possible FTO function, namely, as an "amino-acid sensor"^39^ or "nutrient sensor"^65^. The intake level of protein and carbohydrates may significantly up-regulate the mRNA level of *FTO*^16^. Here, when the HE diet was applied, the total protein content and amino-acid composition did not show obvious deficits in relation to age and the stage of development, whereas with the LE diet provided only 50% of the required proteins and amino acids. Previous in vitro studies showed that the regulation of *Fto* gene expression occurs in response to the lack of essential amino acids^39^ and may be a reason for altered levels of FTO being more pronounced in pigs fed with low calorie diet. Of course, it is possible that more than one signaling pathway is involved in the regulation of FTO, and that certain amino acids, such as glutamine, cysteine and methionine, preferentially trigger one pathway over another^39^. Likewise, it has been shown that the FTO protein expression may be regulated by glucose and might be involved in the sensing of cellular nutrients^65^.

Overall, the biggest limitation of our study is the relatively small number of animals. However, we strongly believe that obtained results in pig models are much more valuable and reliable than rodent models—the results obtained from pig are much easier to transfer to humans.

## Conclusions

Until recently, the role of the FTO protein was practically unknown, even though *FTO* SNPs have been described as strong genetic factors linked to predisposition to obesity and the development of T2D. Our present work focused on the localization of FTO protein in pig tissues. To extend our knowledge regarding the possible role of FTO protein, we used a pig model, because it is much closer to human physiology than rodent models. We found that FTO protein is rather abundantly expressed throughout the studied palette of porcine tissues. Specifically, we detected: a dominant presence in the cerebellum, salivary gland, and kidney; a minor presence in the pancreas, liver, duodenum, small intestine, thyroid, spleen, and adrenal gland; and nearly undetectable in skeletal muscles and adipose tissue. On the other hand, low levels of FTO protein in some tissues were associated with high expression of FTO protein in specific cells within these tissue, such as pancreatic β-cells. Importantly, for the first time, we identified tissues showing FTO presence in the cytoplasm of cells in vivo. Moreover, this unusual cytoplasmic presence was tissue specific. The cytoplasmic expression suggests that in this compartment, FTO protein may play a different function than in the nucleus, such as modifying nucleoprotein complexes. Interestingly, we did not detect FTO protein in the cell excretions, such as bile and saliva, nor in the blood plasma, indicating that FTO protein probably does not take a part in cell-to-cell communication. Further, our comparison of pigs of various ages, metabolic states, and on different diets provides novel insights into possible involvement of FTO protein in a number of metabolic processes. We showed that FTO expression in tissues depends on the metabolic status and age, although we have not yet explored the molecular mechanisms governing this dependency.

In future, the highly specific pancreatic co-localization of the FTO protein with insulin granules within the β-cells should be investigated. Also, a greater understanding of the role of FTO in tissues with very high FTO expression, such as the salivary gland, cerebellum and kidney is required. This work also gives evidence that not just FTO protein level might be determined by age, diet and metabolic status and, thus, future studies should continue developing this knowledge.

## Materials and methods

### Animals and tissue collection

The protocol was conducted in compliance with the European Union’s regulations concerning the protection of experimental animals. The study protocol was approved by the Local Ethical Committee, Warsaw University of Life Sciences, Warsaw, Poland. A total of 40 male and female pigs (*Sus scrofa domesticus,* crossbreed Polish Landrace 50% ♀ and Duroc 25%, Pietrain 25% ♂) from the university research pig farm were used. The herd was virus-negative for porcine reproductive and respiratory syndrome; mycoplasmosis-negative and rhinitis atrophicans infectiosa suum-negative, as confirmed by serum ELISA tests; and leptospirosis-negative as confirmed by the microagglutination test. All piglets were delivered by multiparous sows at term and were clinically healthy, including the newborns that were defined as intrauterine growth retarded (IUGR). Sows were fed a standard diet for pregnant sows (dry matter (DM) 87.6%, metabolizable energy (ME) 11.35 MJ/kg, and crude protein (CP) 12.9%). After farrowing, the diet was switched to the diet for lactating sows (DM 87.3%, ME 12.93 MJ/kg, CP 17.1%). On postnatal days 3 and 17 (PD 3 and 17), all newborn piglets were injected intra-muscularly with 100 mg iron dextran (FeDex, Ferran100, 10% solution, Vet-Agro, Lublin, Poland). The males were castrated in the first postnatal week. From PD 10, piglets were creep fed ad libitum with pre-starter (DM 88.6%, ME 13.3 MJ/kg, CP 18.3%). Piglets were weaned on PD 28 on a standard commercial starter diet (DM 88.6%, ME 13.2 MJ/kg, CP 18.1%). The feed and water were provided ad libitum.

In the first study, a total of 26 pigs from four litters were used. Pigs were weaned on PD28. Six weeks after birth (i.e., on PD42), pigs were divided into three groups. The first group (Cont, n = 6) continued to be fed with a starter diet, then with grower (PD 56–120: DM 88.6%, ME 13.0 MJ/kg, CP 17.0%), grower2 (PD 120–180: DM 88.5%, ME 12.7 MJ/kg, CP 16.0%), and finisher (PD 180–320: DM 88.2%, ME 12.4 MJ/kg, CP 15.0%) diets. The second group was fed with a low energy diet (LE, 50% of the nutritional energy, n = 6). The third group was fed with a high energy diet (HE, 150% of the nutritional energy, n = 14). The increase of energy in the HE group was achieved by the addition of sucrose and rape oil to the standard diet. Animals were adapted to the LE and HE diets over 2 weeks. The diets were continued for nine months, until slaughter. Within this time, 10 out of 14 HE pigs developed insulin resistance, as confirmed by preprandial measurements of blood glucose, triglyceride concentrations, and oral glucose tolerance tests. The six HE pigs manifesting insulin resistance were used in this study.

In the second study, seven pairs of littermate neonates were chosen, each pair from a different litter. In each pair, one piglet was of normal body weight at birth (NBW), i.e., representing the average weight of all littermates (range between 1.3 and 1.6 kg), and the second piglet was of low birth weight (range between 0.6 and 0.9 kg), recognized as asymmetric IUGR^66^ with spontaneous background. The NBW and IUGR piglets were kept together with their litters and were fed by the sow until the slaughter on postnatal day seven (PD7).

Pigs from the first and second study were killed by barbiturate overdose (Morbital, Biowet Puławy Sp. z o.o, Puławy Poland) and exsanguinated. Tissue samples were immediately collected from the brain, liver, spleen, pancreas, small intestine, left submandibular salivary gland, left adrenal gland, left kidney, thyroid, bile, subcutaneous and visceral adipose tissue, and left muscle quadriceps and frozen in liquid nitrogen or fixed in buffered formalin.

### *FTO* SNPs sequencing

Genomic DNA (gDNA) was isolated from livers using the Phenol–Chloroform Isopropanol (PCI, Sigma-Aldrich) DNA Extraction protocol. PCI was added to the sample, vortexed and centrifuged for 10 min at 30,000×*g*. The supernatant was placed in a fresh tube and the CI was added. DNA was precipitated with isopropanol, rinsed with 70% ethanol, and resuspended in distilled water. PCR primers (forward: 5′-TGGCAGCTGAAATACCCTAA-3′, 5′-GACGGGAACCTTGGATTACA-3′; reverse: 5′-CTTCACAGCTATAATTGTAC-3′, 5′-AAGCCCTTACCTCGTTGTGG-3′) were used to amplify exons 3 and 6 of the *FTO* gene using 500 ng of gDNA and MARATON polymerase (A&A Biotechnology). The PCR protocol was as follows: denaturation for 5 min at 95 °C; 30 cycles of 30 s at 95 °C, 30 s at 55 °C and 60 s at 68 °C; the final extension step lasted 10 min at 68 °C. The obtained products were subjected to sequencing (Oligonucleotides Synthesis and DNA Sequencing Laboratory, IBB) with the aforementioned primers. The sequencing results were analyzed with Geneious 10.2.6 software^67^.

### Immunofluorescence and histology studies

After paraffin fixation, tissue samples were sliced into 5 µm sections and rehydrated. For the histology studies, a standard hematoxylin and eosin staining protocol was used. Analysis was performed using light microscope (Olympus BX43) with software cellSens Stanard 1.9. For the immunofluorescence studies, antigen retrieval was performed by boiling the slides in citrate buffer. Non-specific binding was blocked with 1% BSA (Sigma-Aldrich) in PBS at room temperature for 1 h. For the analysis, the primary antibody against FTO (Santa-Cruz Biotechnology sc-271713) was used at a 1:50 ratio with 0.1% BSA in PBS and the slides were incubated overnight at 4 °C. The secondary antibody (Thermo Fisher Scientific AlexaFluor 568, A-11011) was applied at room temperature for 1 h at a dilution of 1:200. Cell nuclei were stained with Hoechst 33342 (Life Technologies) at 10 μg/ml for 30 s at room temperature. Insulin was identified using a primary antibody (Santa Cruz, sc-8033) and visualized by a secondary antibody labeled with Alexa Fluor 488 (Thermo Fisher, A-11059). The slides were mounted in Fluoromount Aqueous Mounting Medium (Sigma Aldrich). For each set of tissue types, a negative control for the analysis of nonspecific signals was performed following the same protocol, but without addition of the primary antibodies. Sequence scanning was used to omit cross-talk between fluorescent dyes. Confocal microscopy (Olympus FV500) with software Fluoroview v5 was employed to obtain quality images. The basic microscope settings were as follows: scan = 200 μm; Kalman = 8; offset = 0%; scanning: sequential for each channel: for Alexa Fluor 488—blue laser (Ar 488 nm); for Alexa Fluor 568- green laser (HeNe 543 nm); for Hoechst 33342 (UV Ar, 351 nm). For the in-tissue cytometry and mapping, whole cross-sections of 100 fields of view (10 × 10, objective 20x) were scanned and the fluorescence intensity of the stained cell nuclei and the expression of FTO protein were measured for each cell. Data were analyzed with scanning cytometry system (SCAN^R, Olympus Poland) with the cell nucleus as a reference point, as described previously^68,69^.

### Tissue samples and Western blot analysis

Frozen samples were homogenized in liquid nitrogen and extracted with RIPA buffer (Sigma-Aldrich) supplemented with 50 mM EDTA and 4 mM PMSF in the presence of protease inhibitor cocktail (Sigma-Aldrich). Cellular debris were spun down and the supernatant’s protein content was measured using the Bradford assay (Bio-Rad). Samples were diluted with SDS-PAGE loading buffer to a protein concentration of 2.5–5 μg/μl and 10 μl portions were loaded onto Mini-PROTEAN TGX 4–15% gradient gels (Bio-Rad). The western blot analysis was performed with specific primary monoclonal antibodies against FTO protein (Santa Cruz Biotechnology), used at dilutions of 1:500, and with the appropriate 1:2000 diluted secondary anti-mouse IgG antibody (Sigma-Aldrich) conjugated with horseradish peroxidase. All incubations were performed in 5% milk/PBST (PBST—phosphate buffer saline with 0.1% TWEEN-20). Chemiluminescence was measured using the ChemiDoc MP Imaging System (Bio-Rad) and ImageLab, version 5.0 build 18 software (https://www.bio-rad.com/en-pl/product/image-lab-software?ID=KRE6P5E8Z). The total protein content was standardized using four steps: (1) equal amounts of tissue were taken for extraction in RIPA buffer (50 mg); (2) the extracts were assayed for protein content by the Bradford method; (3) equal amount of the extracts were loaded onto gels and the amounts were verified by Stain-free staining using ImageLab software; (4) the proteins, transferred to nitrocellulose membranes, were visualized by Ponceau-S reversible staining prior to the final Western blot. Primary monoclonal anti-FTO antibodies were verified by performing silencing of FTO expression in HeLa cell line, using small interfering RNA (siRNA) and subsequent analysis by the Western blot method. After using siRNA, we observed a very week signal of the FTO protein, corresponding to a molecular weight of ~ 58 kDa, as compared to control. Samples were standardized using total protein^70^. The sensitivity of the detection method was 4 ng per sample.

### RNA interference of FTO and cell culture

To validate the quality of antibodies, RNA interference was performed on HeLa cell lines using the lipofectamine RNAiMAX reagent (Invitrogen) and ready-to-use siRNA mixture of *FTO* genes (Santa Cruz Biotechnology). 1 × 10^6^ cells were seeded onto 100 mm diameter dishes. HeLa cells were cultured in DMEM medium (Life Technology) with 10% fetal bovine serum (Life Technology) and 0.1% antibiotics (penicillin/streptomycin, Life Technology). Cells were grown in a humidified atmosphere of CO2/air (5/95%) at 37 °C. The HeLa cell line was provided by Culture Collection, Public Health England, Porton Down, Salisbury, UK (Cat. No.: 93021013). The siRNAs mixtures were added after a 24 h incubation; after 48 h, the cells were collected for Western blot analysis. Adherent cells were scraped and centrifuged (800×*g*, 10 min, 4 °C). Next, the cells were resuspended in RIPA buffer with a 1 × mammalian protease inhibitor cocktail (Sigma-Aldrich). The lysates were spun down at 30,130×g and supernatants were assayed for protein content using the Bradford assay. 20 μg of each sample was taken for Western blot analysis. The obtained FTO bands were quantified by densitometry using ChemiDoc MP Imaging System (Bio-Rad).

### Statistical analysis

The results were subjected to three-stage statistical analysis. In the first step, we checked the uniformity of the SD and performed the Kolmogorov–Smirnov test of the normal distribution. In all examined groups, there were no indications for exclusion based on the assumption of normality of distribution. Next, the MANOVA test was performed to check if there are statistical differences in FTO levels among all tissues. A further ANOVA test with Benjamini–Hochberg adjustment was used to evaluate the tissues that differed between the groups. The data were then analyzed with an unpaired *t* test with Benjamini–Hochberg adjustment. All analyses were performed using the software R (version 3.3.0, www.r-project.org) with ‘cowplot’, ‘scales’, ‘stats’, ‘gridExtra’, ‘outliers’ and ‘ggplot2′ packages^71–76^. The significance level α of 0.05 was used for all statistical tests.

### Ethics approval and consent to participate

The protocol was conducted in compliance with the European Union regulations concerning the protection of experimental animals. The study protocol was approved by the Local Ethical Committee, Warsaw University of Life Sciences, Warsaw, Poland No. 7/2015 and No. 8/2015.

## Supplementary information

Supplementary Information 1.
