# Supplementary material for: Intracellular and tissue specific expression of FTO protein in pig: changes with age, energy intake and metabolic status
Source: Sci Rep. 2020 Aug 3;10:13029. doi: 10.1038/s41598-020-69856-5 (PMC7400765; doi:10.1038/s41598-020-69856-5)
Supplement: Supplementary file 1 — Supplementary Information 1. [file 41598_2020_69856_MOESM1_ESM.docx]

**Supplement**

**Intracellular and tissue specific expression of FTO protein in pig - changes with age, energy intake and metabolic status**

Karolina Ferenc^1*^, Tomaš Pilžys^2*^, Damian Garbicz^2^, Michał Marcinkowski^2^, Oleksandr Skorobogatov^2^, Małgorzata Dylewska^2^, Zdzisław Gajewski^1^, Elżbieta Grzesiuk^2#^, Romuald Zabielski^1#^

^1^Veterinary Research Centre, Department of Large Animal Diseases and Clinic, Institute of Veterinary Medicine, Warsaw University of Life Sciences, Nowoursynowska 100, 02-797 Warsaw, Poland, ^2^Institute of Biochemistry and Biophysics, Polish Academy of Sciences, Pawińskiego 5a, 02-106 Warsaw, Poland


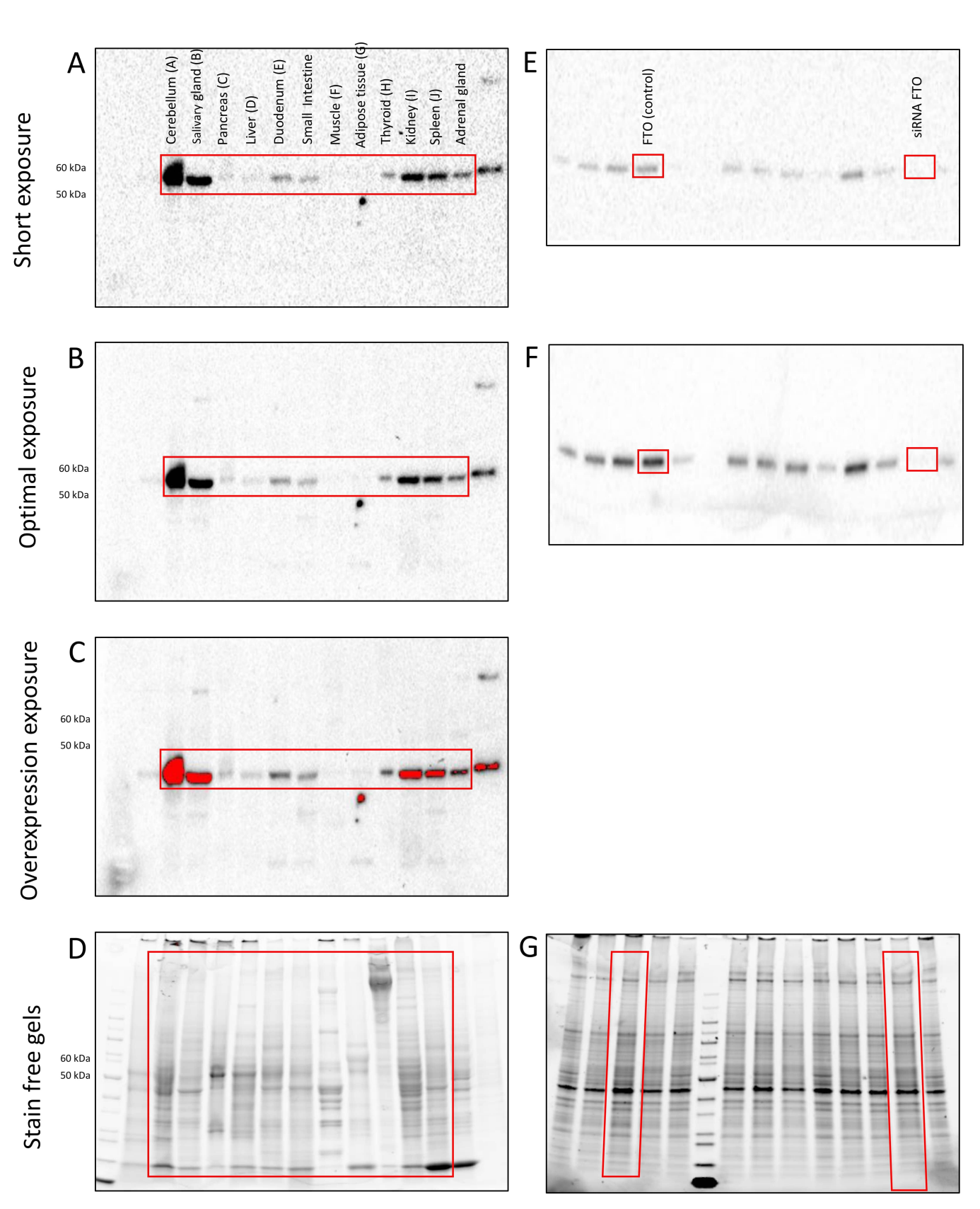


**Figure S1. Western blot analysis of FTO protein.** (A, B, C) FTO expression in various tissues; (E, F) RNA interference verification; FTO (control) – HeLa cells not treated by siRNA; siRNA FTO – Hela cells treated with siRNA of FTO. (D, G) Stain free gels for corresponding blots. (Image Lab, version 5.0 build 18, ChemiDoc MP Imaging System)


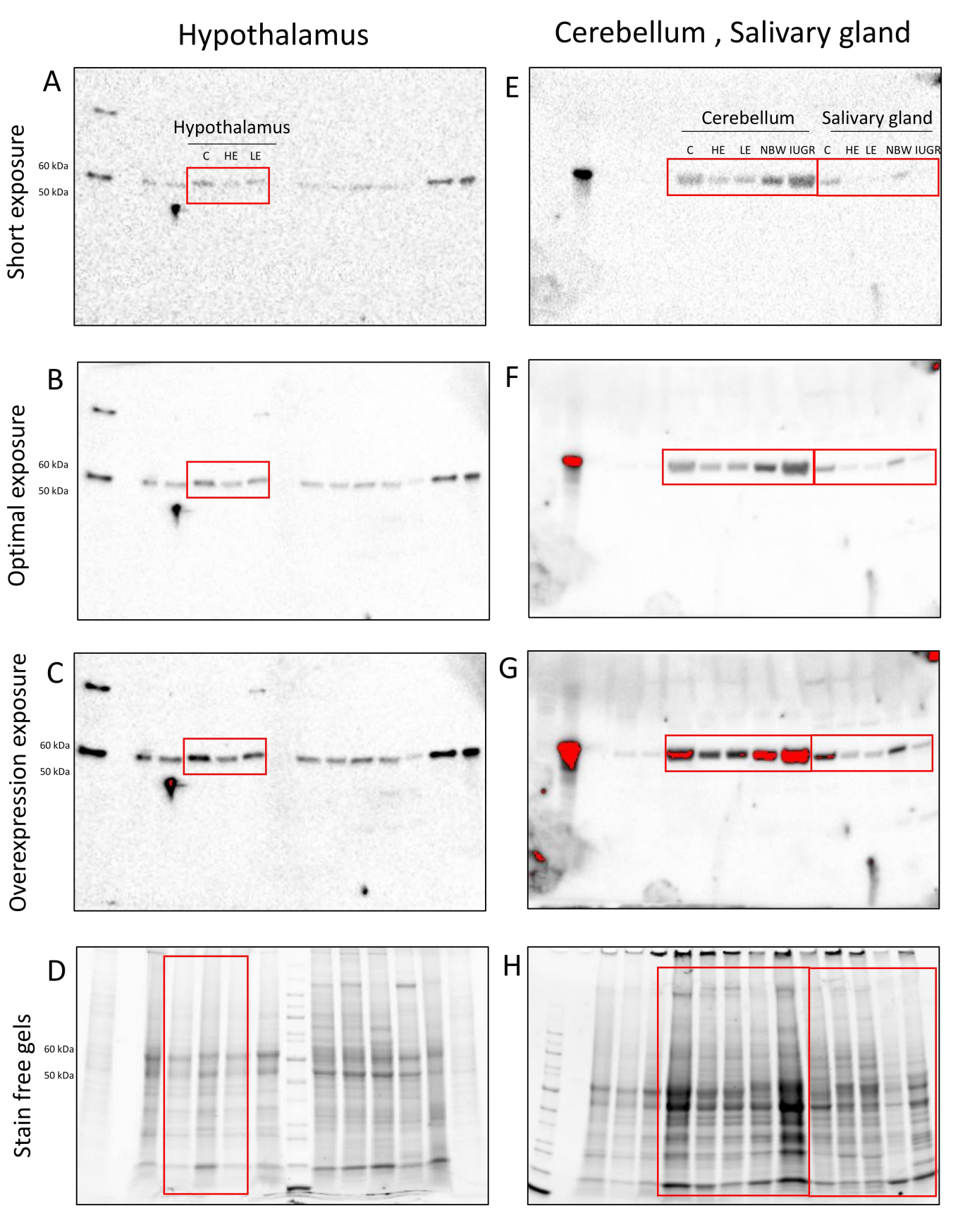


**Figure S2. Western blot analysis of FTO protein.** (A, B, C) FTO expression in hypothalamus; C – control, HE – High Energy diet; LE – Low Energy diet. (E, F, G) FTO expression in cerebellum, salivary gland; C – control, HE – High Energy diet; LE – Low Energy diet; NBW – normal body weight, IUGR – intrauterine growth restriction. (D, H) Stain free gels for corresponding blots. (Image Lab, version 5.0 build 18, ChemiDoc MP Imaging System)


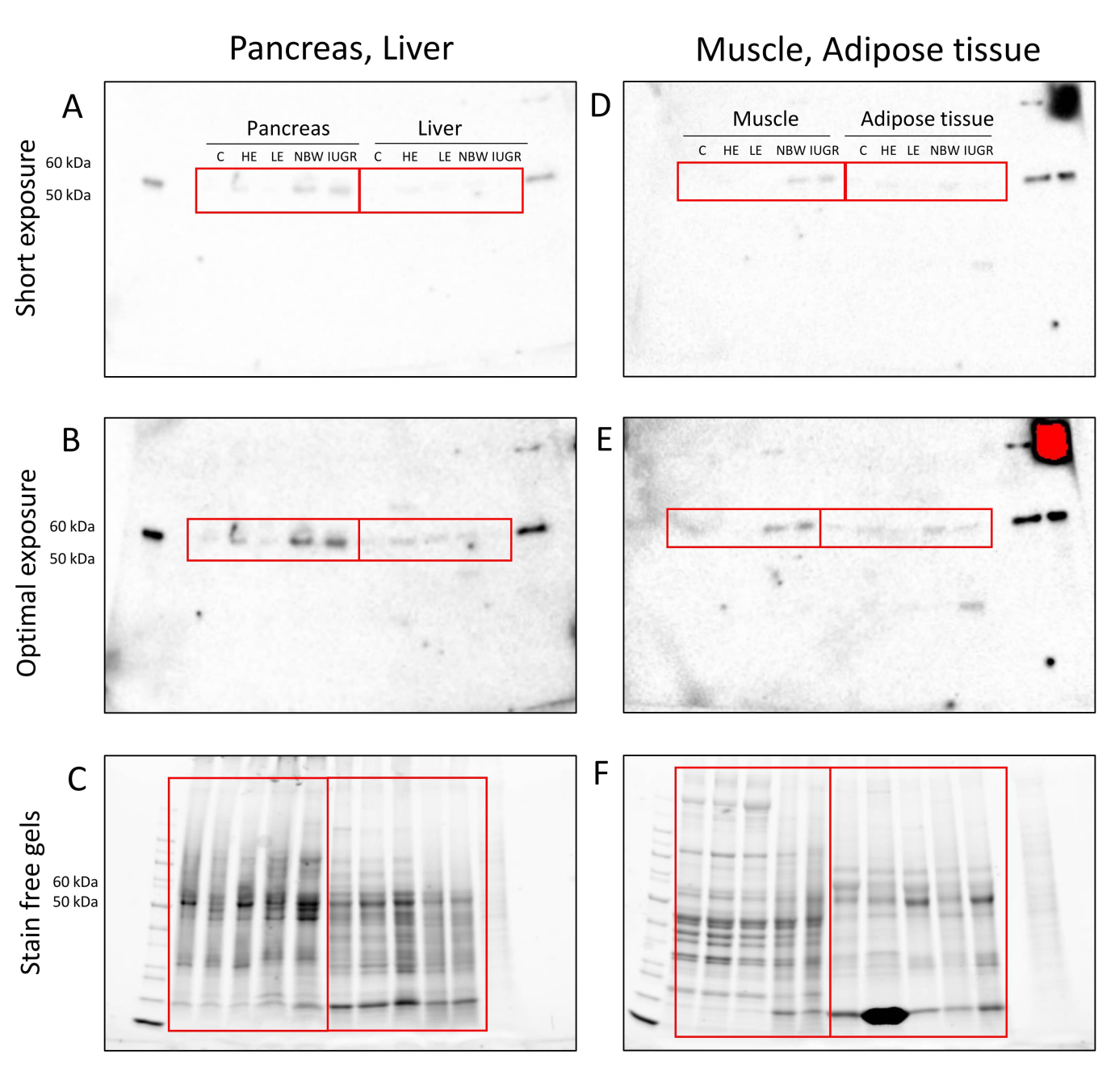


**Figure S3. Western blot analysis of FTO protein.** (A, B) FTO expression in pancreas, liver; C – control, HE – High Energy diet; LE – Low Energy diet; ; NBW – normal body weight, IUGR – intrauterine growth restriction. (D, E) FTO expression in muscle, adipose tissue; C – control, HE – High Energy diet; LE – Low Energy diet; NBW – normal body weight, IUGR – intrauterine growth restriction (C, F) Stain free gels for corresponding blots. (Image Lab, version 5.0 build 18, ChemiDoc MP Imaging System)


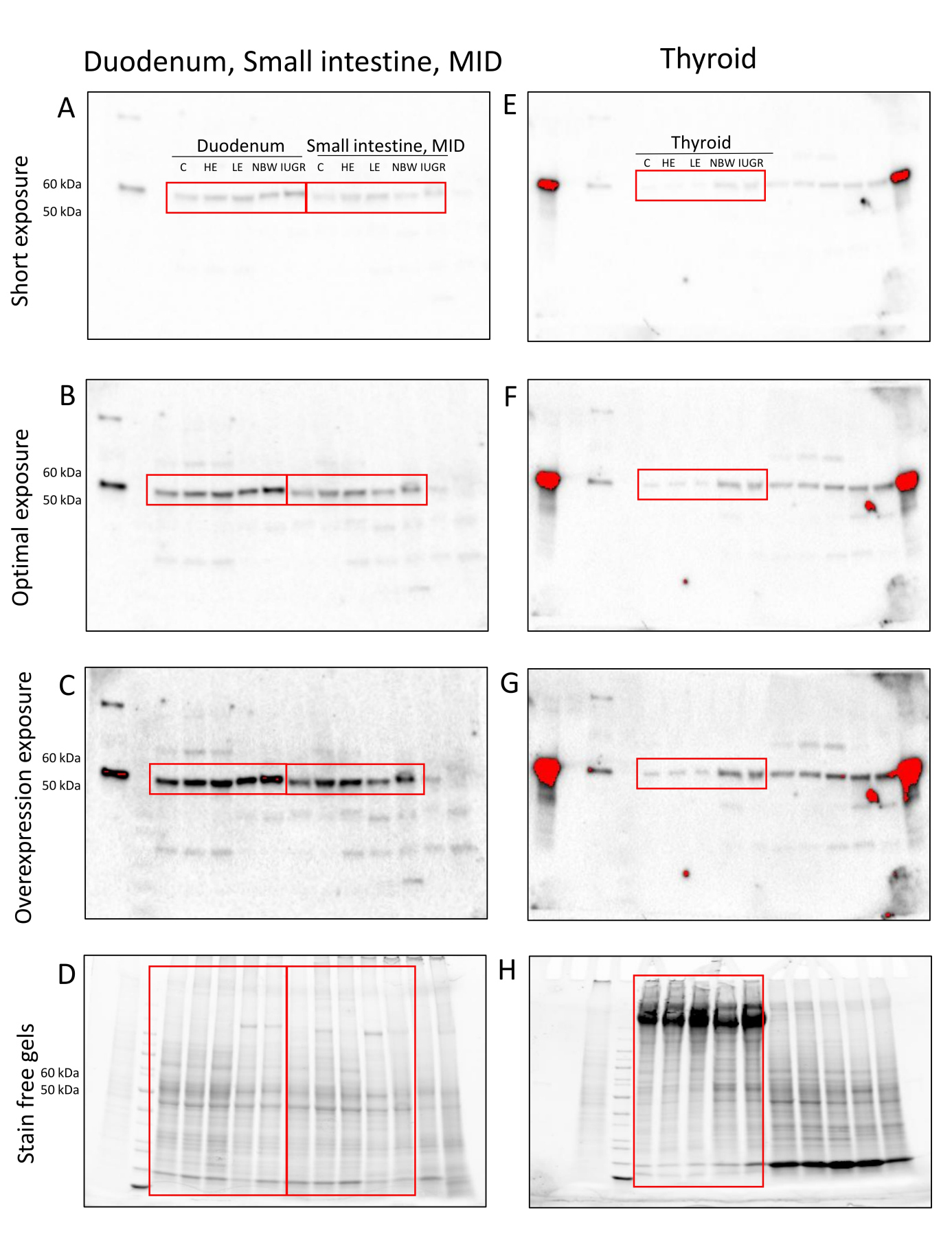


**Figure S4. Western blot analysis of FTO protein.** (A, B, C) FTO expression in duodenum, small intestine, MID; C – control, HE – High Energy diet; LE – Low Energy diet; NBW – normal body weight, IUGR – intrauterine growth restriction. (E, F, G) FTO expression in thyroid, salivary gland; C – control, HE – High Energy diet; LE – Low Energy diet; NBW – normal body weight, IUGR – intrauterine growth restriction. (D, H) Stain free gels for corresponding blots. (Image Lab, version 5.0 build 18, ChemiDoc MP Imaging System)


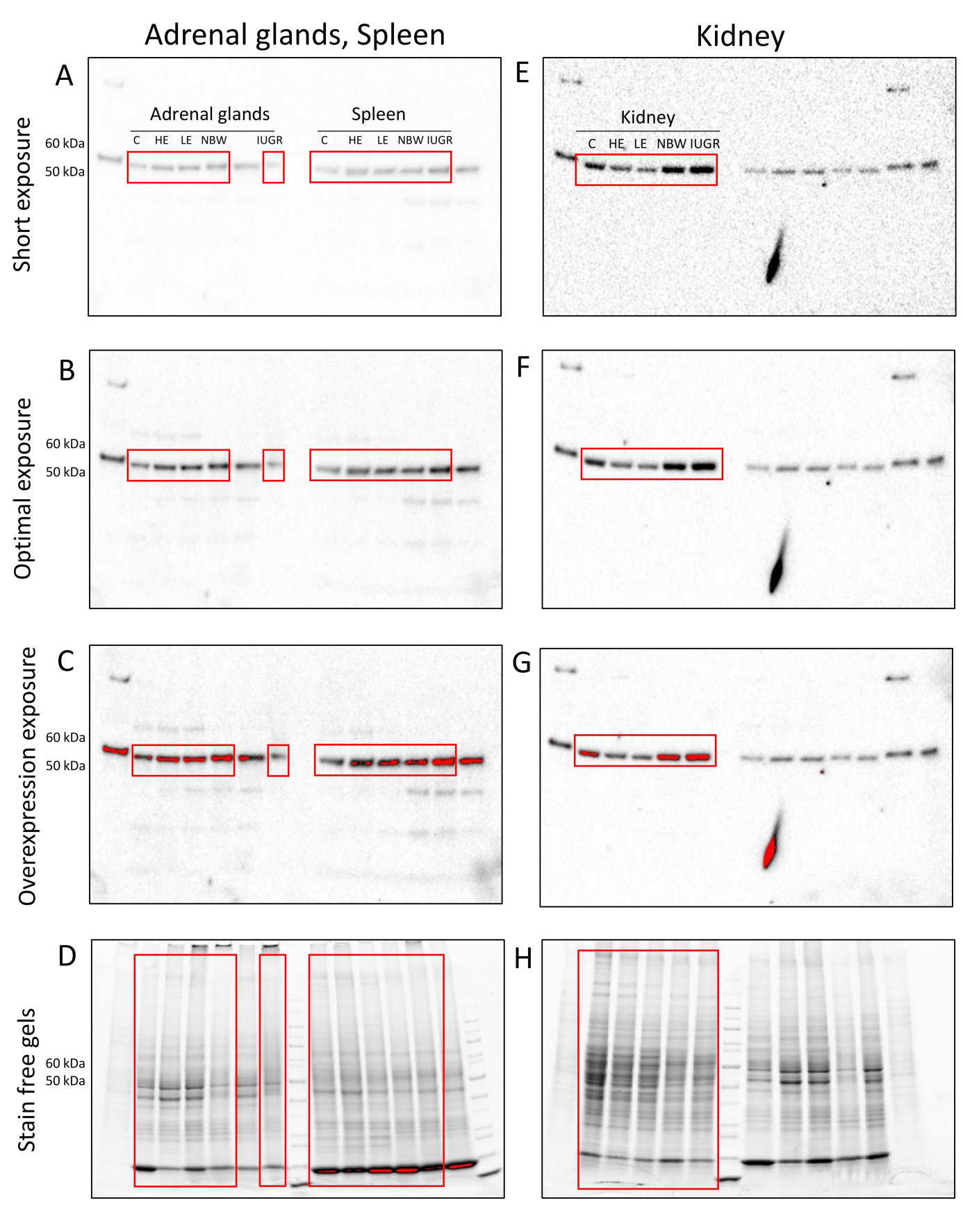


**Figure S5. Western blot analysis of FTO protein.** (A, B, C) FTO expression in adrenal gland, spleen; C – control, HE – High Energy diet; LE – Low Energy diet; NBW – normal body weight, IUGR – intrauterine growth restriction. (E, F, G) FTO expression in kidney, salivary gland; C – control, HE – High Energy diet; LE – Low Energy diet; NBW – normal body weight, IUGR – intrauterine growth restriction. (D, H) Stain free gels for corresponding blots. (Image Lab, version 5.0 build 18, ChemiDoc MP Imaging System)
